# Study of Si and Ge Atoms Termination Using H-Dilution in SiGe:H Alloys Deposited by Radio Frequency (13.56 MHz) Plasma Discharge at Low Temperature

**DOI:** 10.3390/ma13051045

**Published:** 2020-02-26

**Authors:** Ismael Cosme, Andrey Kosarev, Saraí Zarate-Galvez, Hiram E. Martinez, Svetlana Mansurova, Yuri Kudriavtsev

**Affiliations:** 1 Instituto Nacional de Astrofísica, Óptica y Electrónica (INAOE), Luis Enrique Erro # 1, Tonantzintla, Puebla 72840, Mexico; akosarev@inaoep.mx (A.K.); sarai.zarate@inaoep.mx (S.Z.-G.); hiram@inaoep.mx (H.E.M.); smansur@inaoep.mx (S.M.); 2 Consejo Nacional de Ciencia y Tecnología—INAOE, Luis Enrique Erro # 1, Tonantzintla, Puebla 72840, Mexico; 3 Centro de Investigación y Estudios Avanzados (CINVESTAV), Departamento de Ingeniería Eléctrica, Cinvestav IPN, Ciudad de Mexico 07360, Mexico; yuriyk@cinvestav.mx

**Keywords:** Alloys, silicon-germanium, hydrogen dilution, RF PECVD

## Abstract

In this work, we present the study of the atomic composition in amorphous Si_X_Ge_Y_:H_Z_ films deposited by radio frequency (RF—13.56 MHz) plasma discharge at low deposition temperature. A study and control of Si and Ge atoms termination using H-dilution in SiGe:H alloys deposited by RF plasma discharge was conducted and we made a comparison with low-frequency plasma discharge studies. Solid contents of the main elements and contaminants were determined by SIMS technique. It was found that for low dilution rates from R_H_ = 9 to 30, the germanium content in the solid phase strongly depends on the hydrogen dilution and varies from Y = 0.49 to 0.68. On the other hand, with a higher presence of hydrogen in the mixture, the germanium content does not change and remains close to the value of Y = 0.69. The coefficient of Ge preferential incorporation depended on R_H_ and varied from P_Ge_ = 0.8 to 4.3. Also, the termination of Si and Ge atoms with hydrogen was studied using FTIR spectroscopy. Preferential termination of Si atoms was observed in the films deposited with low R_H_ < 20, while preferential termination of Ge atoms was found in the films deposited with high R_H_ > 40. In the range of 20 < R_H_ < 40, hydrogen created chemical bonds with both Si and Ge atoms without preference.

## 1. Introduction

Silicon-germanium thin films (Si_X_Ge_Y_:H_Z_) deposited by Plasma Enhanced Chemical Vapor Deposition (PECVD) have been studied over the last decades regarding solar cell applications due to their reduced band-gap and their absorption in the infrared spectrum (IR) [1]. In recent years, silicon-germanium alloys have also demonstrated potential applications in new device concepts such as 3D structured devices [2,3,4], un-cooled micro-bolometers [5], microelectromechanical systems (MEMS) [6], and biomedical applications [7]. However, the increase of Ge content in these alloys is reported to deteriorate the electronic properties and the device characteristics. Then, better performance and stability requires much effort to improve the film quality.

One promising technique to improve the film quality of Si_X_Ge_Y_:H_Z_ alloys deposited by PECVD is the hydrogen dilution method [8,9]. Many groups have reported results obtained by this technique and have discussed the role of hydrogen dilution for a relatively low hydrogen dilution ratio R_H_ in the range of less than R_H_ = 20 (R_H_ = Q_H2_/Q_SiH4_, where Q_H2_ is the hydrogen flow rate, and Q_SiH4_ is the silane flow rate) at substrate temperatures above 200 °C [10]. Most of the papers deal, however, with relatively low Ge concentration (Y < 0.5) and high deposition temperatures (Td > 200 °C) because these parameters provide films with optoelectronic properties acceptable for application in the device structures.

The investigation of Si_X_Ge_Y_:H_Z_ films over the entire range 0 < Y < 1 was systematically studied by our group for low frequency (LF) and high deposition temperature, and the results were reported in [9]. Good quality SiGe films were reported also for LF PECVD in references. [11,12,13]. On the other hand, the effect of dilution gas at low deposition temperatures (T_d_ < 200 °C) on both, film growth and electronic properties has not been systematically studied for standard radio frequency (13.56 MHz) conditions. However, nowadays thin films deposited at low temperatures (Td < 200 °C) are a requirement for novel device applications such as hybrid inorganic-polymer structures [14,15] and flexible substrates [16].

It should be noted that the problem of optimization of conditions for PECVD fabrication of semiconductor materials consisting of more than one semiconductor atom e.g., Si_X_Ge_Y_:H_Z_ is more complex than that for one semiconductor atom, e.g., Si:H. The deposition of hydrogenated alloys has two important aspects: (a) Incorporation of semiconductor atoms from the gas phase (typically preferential incorporation of one atom is observed with the preferential factor depending on the deposition conditions), and (b) hydrogen termination of both atoms, let us say Ge–H and Si–H; hydrogen termination is also realized with the preference of one atom for hydrogen termination. The latter has not been studied and therefore the optimal (from point of view electronic properties) hydrogen distribution between two atoms is unknown. Hydrogen dilution of the gas mixture used for semiconductor deposition has demonstrated a significant effect on deposition rate, atom incorporation from the gas phase into a solid-state, and as expected on hydrogen termination of both atoms.

This paper reports the composition and Si and Ge atoms termination in Si_X_Ge_Y_:H_Z_ films using hydrogen dilution in deposition under standard RF (13.56 MHz) PECVD at low deposition temperature (T_d_ = 160 °C) compatible with polymer semiconductor and flexible plastic substrates [17]. The effect of hydrogen dilution on growth, composition, and Si–H and Ge–H hydrogen bonding of high Ge content Si_X_Ge_Y_:H_Z_ (Y > 0.5) was studied.

## 2. Materials and Methods

**Figure A1:**
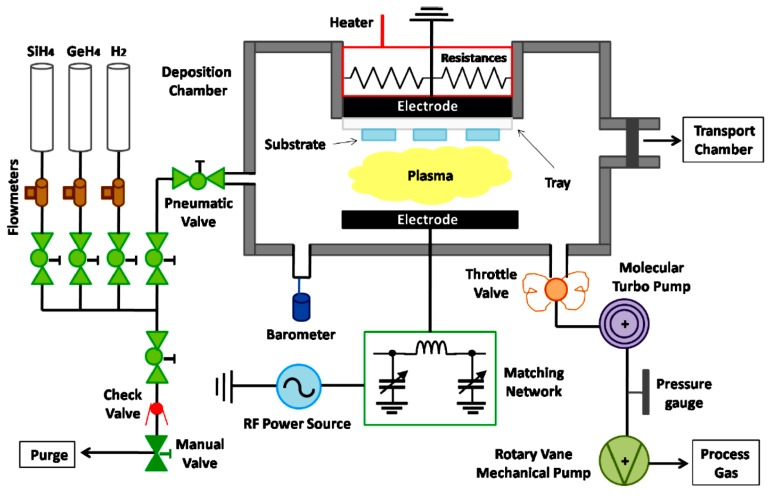
MVSystem Plasma Enhanced Chemical Vapor Deposition (PECVD) chamber configuration.

The samples were fabricated using a cluster tool system from “MVSystem. Inc. (Denver, CO, USA)” in a standard capacitive PECVD chamber (see Appendix A, Figure A1). The R.F. electrode assembly consists of a dark shield, cathode- anode electrode configuration with a distance of 1.9 cm, and automatic matching. The gas injector contains multiple small holes and the location provides a uniform flow of gasses across the plasma region with a substrate area of 15.6 × 15.6 cm^2^. SiGe:H films were deposited in the chamber after cleaning and passivation of the walls by the growth of intrinsic film, the background vacuum level was lower than 5 × 10^−7^ Torr and a leakage rate was R_leak_ = 4.5 × 10^−4^ sccm. Intrinsic SiGe:H films were deposited from (50% SiH_4_ + 50% GeH_4_) + H_2_ mixture, all the gases used were semiconductor purity and were pre-mixed before introducing them to the chamber. The hydrogen dilution ratio was defined as R_H_ = C_H_/(C_Si_ + C_Ge_) where C_H_ is the concentration of hydrogen atoms, C_Si_ and C_Ge_ are the concentrations of Si and Ge atoms in the gas phase, respectively. Thus, R_H_ was varied from 9 to 80 by increasing the H_2_ flow from Q_H2_ = 9 to 80 sccm at constant pressure P = 0.55 Torr. Deposition temperature was fixed at T_d_ = 160 °C and deposition time was set at t_d_ = 2000 s. RF discharge was excited at frequency f = 13.56 MHz with power W = 5 W (22 mW/cm^2^). The substrates used were p^+^-doped c-Si wafers for SIMS analysis. The experimental films were characterized by the measurements as follows: deposition rate, V_d_, was obtained from step profile measurements of the silicon–germanium films with a “DektakXT profiler” (Bruker, Billerica, MA, USA). The deposition rate was calculated assuming that the film thickness is a linear function of time: V_d_ = d/t_d_, where d is the film thickness and t_d_ is the deposition time.

Solid-phase atomic content in SiGe:H films was determined by Secondary Ion Mass Spectroscopy (SIMS) Technique. For this purpose, a time of flight TOF-SIMS-5 instrument from “ION TOF GmbH” (Muster, Germany) was used. The depth profiling was realized with a double beam regime: A pulsing Bi^+^ ions beam was used for analysis, and low energy Cs^+^ ions beam for a delicate sputtering. Both negative secondary ions and positive CsM^+^ cluster ions (where M is the element of interest) were monitored in parallel measurements for dopants, contaminants (C, O, N, F), and the main components (Si, Ge, H) characterization. Quantification of intensity in experimental data was performed using the implanted standards and by measurements of reference SiGe compounds with a known composition.

H-termination characterization was performed, the infrared (IR) absorption spectra of the films were measured with an FTIR spectrometer from “Brucker Optics” (model “Vector-22”, Ettingen, Germany) over the range 350–4000 cm^−1^. The measured absorbance spectra were normalized to the absorbance spectrum of a crystalline silicon substrate, and the spectral absorption spectra were calculated. The spectra were analyzed systematically after baseline subtraction and computer deconvolution.

## 3. Results

### 3.1. Film Growth

**Figure 1:**
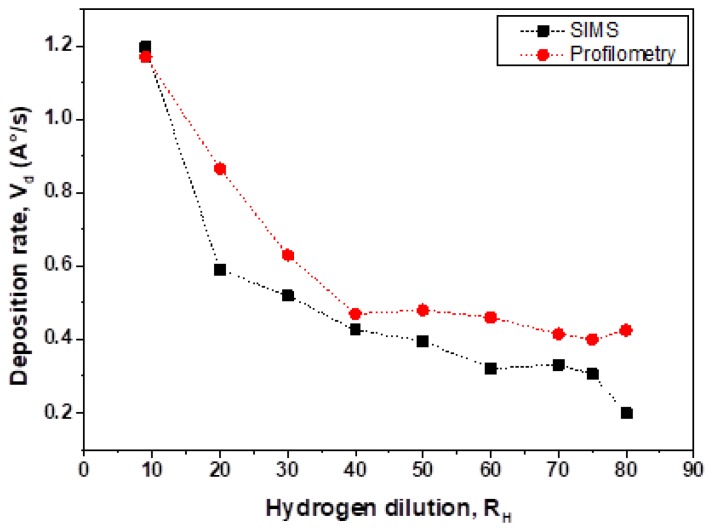
Deposition rate as a function of hydrogen dilution ratio V_d_(R_H_) calculated from step measurements and estimation of depth from measurements of the crater created by SIMS.

After fabrication, the thickness of the films was measured by the profiler to calculate the deposition rate V_d_. The deposition rate of film thickness is depicted in Figure 1 in comparison with that obtained from the measurements of the crater generated by SIMS measurements. V_d_(R_H_) shows a reduction from V_d_ = 1.2 to 0.5 A/s with a change in R_H_ from R_H_ = 9 to 40 and only a small change (practically negligible) in the range R_H_ = 40 to 80. Comparing these V_d_ values with those reported in reference [9] for SiGe:H films deposited by low frequency (LF) PECVD and high temperature we noticed similar V_d_ values for low Ge content (about 0.1 in the gas phase) to those for RF PECVD films. On the other hand, values of the deposition rate of V_d_ = 0.9 ± 0.1 Å/s are found in reference [18] for film growth with 0.5 Ge content in the gas phase. It is important to note that V_d_ behavior depends on the selection of experimental variables, here the hydrogen dilution provides lower deposition rates when it is raised. However, opposite behavior is found when the germanium content in gas phase C_G_ is taken as the variable e.g., the deposition rate increases from 1.5 to 3.7 Å/s for LF discharge [9] and from 4 to 11 Å/s for RF discharge when C_Ge_ is raised, for hydrogen dilution ratios of R_H_ = 0.4 and R_H_ = 20, respectively.

### 3.2. Composition of the Films

**Figure 2:**
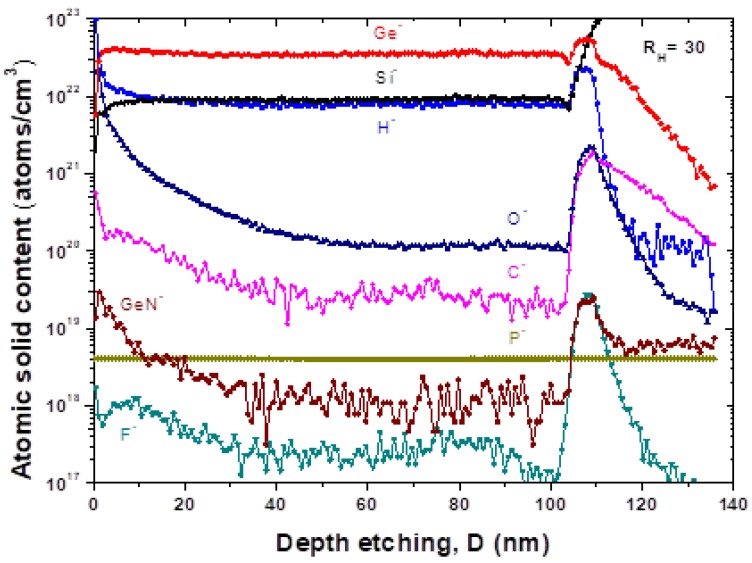
SIMS depth profile of H-, C-, O-, F-, Si-, P-, Ge-, GeN- elements in silicon–germanium film (R_H_ = 30) grown on p^+^-doped c-Si wafer.

A typical SIMS profile is shown in Figure 2 corresponding to a Si_X_Ge_Y_:H_Z_ film deposited at hydrogen dilution R_H_ = 30. The main contaminant element is seen to be oxygen. However, the SIMS signal also contains a contribution from residuals in the analytical chamber of the SIMS instrument. The contents of Oxygen (O), Carbon (C), and Nitrogen (N) atoms in the solid films are around the level of 2.8 ± 0.9 × 10^20^, 4 ± 1 × 10^19^, and 1.40 ± 0.05 × 10^19^ atoms/cm^3^, respectively. The reasons for relatively high contaminations are not clear at present and could be caused by both, deposition processes and/or vacuum conditions in the SIMS measurements.

**Figure 3:**
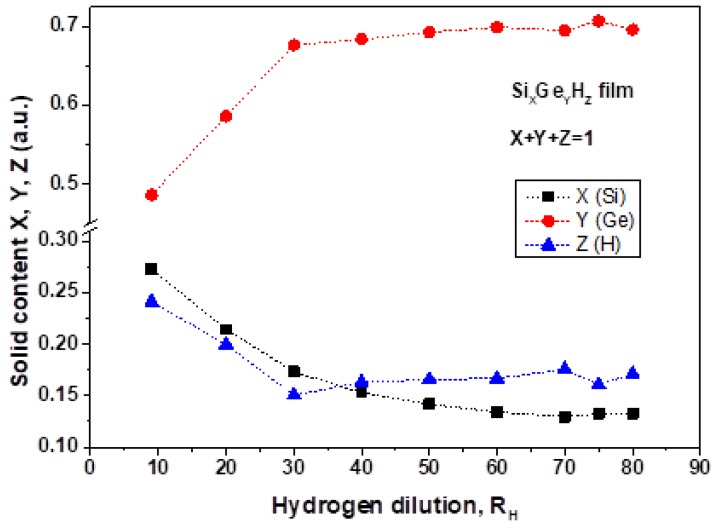
Solid content composition for Si_X_Ge_Y_:H_Z_ films as a function of the hydrogen dilution rate.

The relative contents of X, Y and Z elements as a function of R_H_ in Si_X_Ge_Y_:H_Z_ are presented in Figure 3. Hydrogen content Z in the solid-state in the films decreases from Z = 0.24 to 0.15 when the dilution ratio increases from R_H_ = 9 to 30. Further H-dilution slightly changes the hydrogen content from Z = 0.15 to 0.175. This behavior together with almost constant values of X and Y in this region suggests that there is no change in hydrogen termination of both Si and Ge atoms in the films. Germanium content increases from Y = 0.485 to 0.70 as the dilution ratio increases from R_H_ = 9 to 30. In the range of R_H_ = 30 to 80, the germanium solid content is constant Y = 0.69 ± 0.01. Such behavior and the Y value differ from those in the films grown from LF discharge, in which Y has a constant value of 0.965 ± 0.005 in the entire reported range of R_H_ = 20 to 80. As it is depicted in Figure 3 higher dilution provides higher germanium and lower hydrogen content in comparison with that at low dilution, a similar result is reported in reference [10]. Silicon content decreases from X = 0.27 to 0.13 with R_H_ increase from R_H_ = 9 to R_H_ = 40 and then it remains at a constant value X = 0.137 ± 0.008 from R_H_ = 40 to 80.

**Figure 4:**
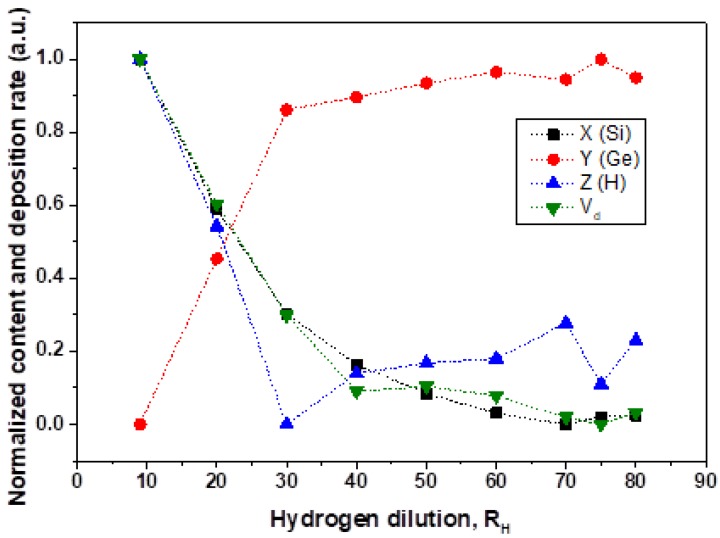
Normalized values for solid silicon, germanium, and hydrogen content and deposition rate of Si_X_Ge_Y_:H_Z_ films as a function of hydrogen dilution ratio.

Normalized solid component values of Si_X_Ge_Y_:H_Z_ films and deposition rates as a function of hydrogen dilution are shown in Figure 4. The deposition rate V_d_(R_H_) has similar behavior to that of the solid content of silicon, meaning that the deposition of the films is controlled by Si atom incorporation. If we compare X(R_H_) and Y(R_H_) with hydrogen behavior Z(R_H_) there would be a reason to suggest that hydrogen mostly terminates Si atoms in the entire studied R_H_ range. In other words, Ge atoms could be not sufficiently passivated by hydrogen which results in deterioration of the electronic properties. However, this suggestion is not supported by FTIR data. We continue the discussion of H-termination in the section related to FTIR data analysis. This aspect has not been systematically reported and analyzed as yet in the literature.

**Figure 5:**
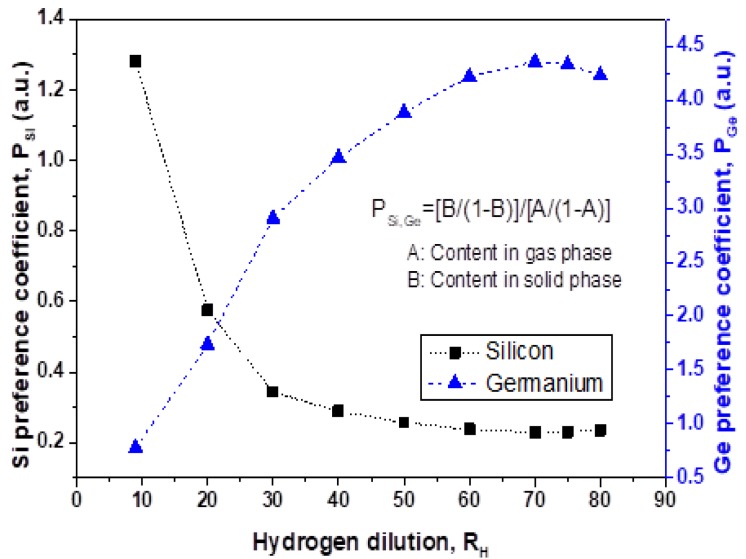
Coefficients of preferential incorporation of Si and Ge atoms, P_Si_ and P_Ge_, as a function of hydrogen dilution.

Incorporation of Ge atoms in Si–Ge:H film from the gas phase can be characterized by the coefficient of preferential incorporation (see e.g., [19]) defined as P_Ge_ = (relative Ge content in solid phase)/(relative content of Ge atoms in gas phase) and P_Si_ = (relative Si content in solid phase)/(relative content of Si atoms in gas phase). In the alloys, the relationship P_Ge_ = 1/P_Si_ is evident. The coefficients of preferential incorporation, P_Si_, and P_Ge_, are shown in Figure 5. For P_Ge_ calculation the germanium content in the gas phase was A = GeH_4_/GeH_4_ + SiH_4_ and solid content is represented by the Si_1−B_Ge_B_:H formula. This coefficient is conventionally obtained as the best fit parameter in experimental data processing for variation of composition within the entire range. Interestingly, many authors reported the best fit of their experimental data (obtained in very different conditions) with only one parameter (P_Ge_) within the entire range of composition. Concrete P_Ge_ values were different depending on deposition conditions. In this work, we calculated P_Ge_, and P_Si_ for Ge–Si:H films deposited with different H-dilution in RF discharge.

Preferential incorporation strongly depends on hydrogen dilution in the range of R_H_ = 9 to 20 for silicon and R_H_ = 9 to 50 for germanium. For R_H_ = 20, P_Ge_ has a lower value of P_Ge_ = 1.73 in comparison with that reported for LF discharge (P_Ge_ = 6.44) [9]. For R_H_ = 9, P_Ge_ has a lower value of P_Ge_ = 0.78 in comparison with that reported for RF discharge (P_Ge_ = 6.1 and 4.6) [18]. The difference in P_Ge_ values could be attributed to differences in fabrication parameters. For example, power density in our experiments was w = 22 mW/cm^2^ in comparison with w = 5 and 80 mW/cm^2^ reported in [18] which provided P_Ge_ = 6.1 and 4.6, respectively. The latter P_Ge_ value is different from P_Ge_ = 12 found for SiGe:H films deposited at relatively high-power density (110 mW/cm^2^) but low flow rate (7 sccm) and low dilution ratio (0.4) reported in [19]. Higher power densities provide the higher germanium content in the solid phase as shown in reference [10] for undiluted SiH_4_ + GeH_4_ mixtures; opposite behavior is observed for diluted mixtures. This data agrees with the high P_Ge_ > 1 value. From R_H_ = 60 to 80, P_Ge_ has a weak dependence on hydrogen dilution providing a value around P_Ge_ = 4.1 which is close to that discussed previously for RF discharge. In the case of other parameters such as deposition temperature or Ge content, there is no evidence of influence on P_Ge_ in the studied range [18].

### 3.3. H-Termination of Si and Ge Atoms

**Figure 6:**
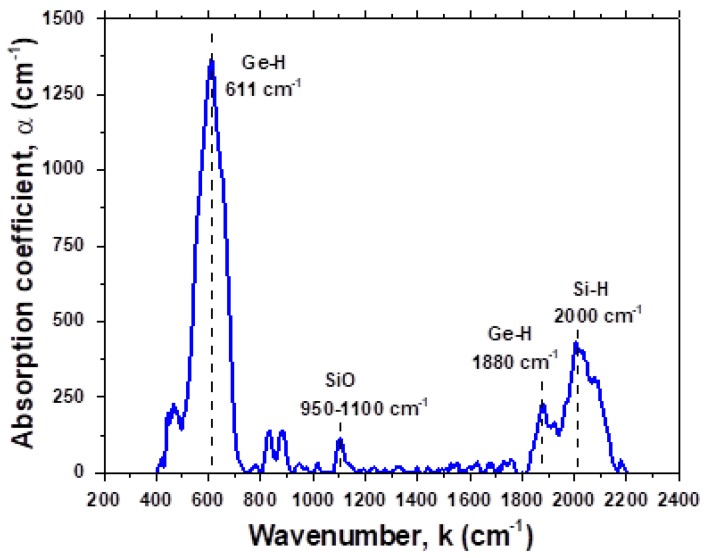
General view of IR spectra in the Si–Ge:H samples deposited at R_H_ = 0.9.

**Figure 7:**
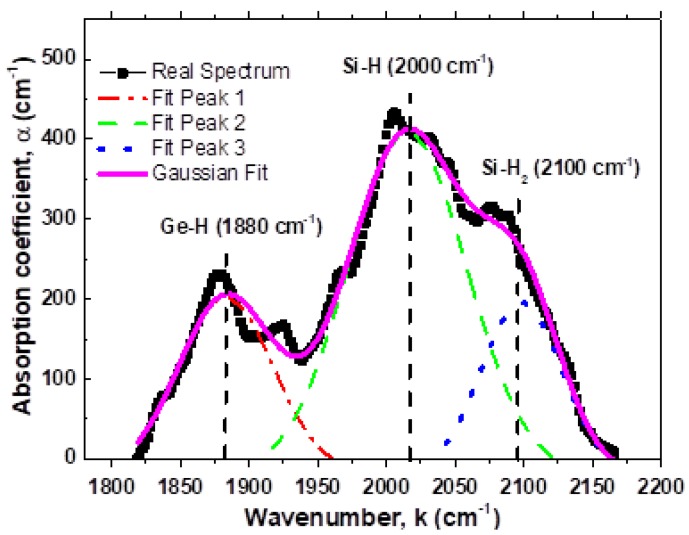
Experimental IR spectrum of stretching vibration modes and deconvolution of Ge–H and Si–H for the SiGe:H films deposited at R_H_ = 09.

Hydrogen bonding with Si and Ge atoms was studied with FTIR spectroscopy. The general view of the FTIR spectrum is presented in Figure 6. Three groups of modes can be distinguished in the Figure: (1) Stretching mode for Si–H, Ge–H bonds (k = 1800–2200 cm^−1^, (2) deformation modes for Si–H and Ge–H bonds (k = 500–700 cm^−1^) and (3) Si–O and Ge–O bonds (k = 1000–1200 cm^−1^, and k = 900–1000 cm^−1^, respectively). We shall focus on the regions of stretching modes for Si–H and Ge–H bonds. For illustration, a fragment of the FTIR spectrum corresponding to Si–H and Ge–H modes is shown in Figure 7.

**Figure 8:**
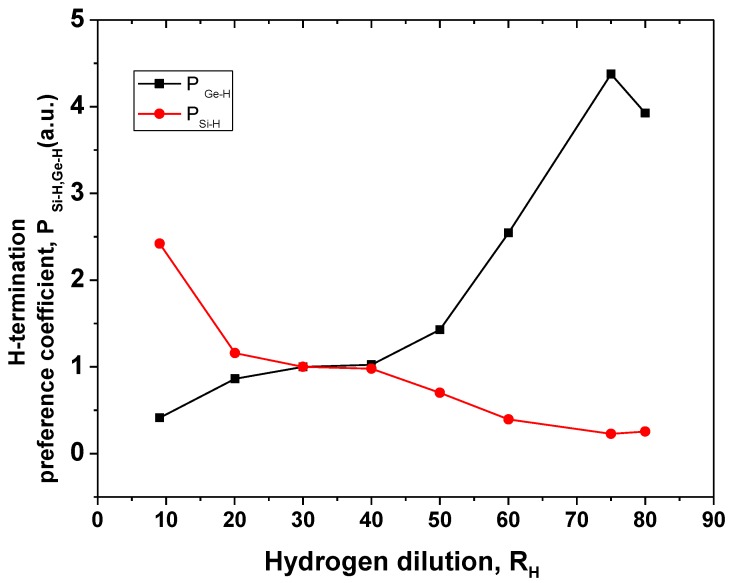
Factor of preferential H- termination for Si–H and Ge–H bonds in the Si–Ge:H films deposited with different H-dilution.

Both, the experimental data and results of the deconvolution are presented in this figure. In this, three peaks are observed around k ≈ 1880 cm^−1^, k ≈ 2000 cm^−1^, and k ≈ 2100 cm^−1^ assigned to Ge–H, Si–H, and Si–H_2_ stretching modes, respectively. In Table A1, the peaks, contour width, and area are summarized. It is possible to observe in Table A1 that the hydrogen dilution changes remarkably these characteristics. Figure 8 shows the plot of the H-termination preference coefficients for Si–H and Ge–H bonds for different H-dilution, where P_Ge–H_ = (relative Ge–H bonds content in solid phase)/(relative content of Ge atoms in gas phase) and P_Si–H_ = (relative Si–H bonds content in solid phase)/(relative content of Si atoms in gas phase); the relative contents were determinate from the IR stretching Ge–H (1880 cm^−1^) and the Si–H (2000 cm^−1^) absorption band integrated areas. The definition and explanation of P_GeH_ y P_SiH_ concepts are similar to P_Ge_ and P_Si_ gave in reference [19]. For low R_H_ ≤ 20 the P_SiH_ decreases from 2.42 to 1.16 and P_GeH_ slightly increases from 0.42–0.57. In the range of 20 ≤ R_H_ ≤ 40 the values are P_GeH_ = P_SiH_ = 1 (both values calculated from stretching modes), this means that the H termination for Si and Ge atoms is similar.

In the range R_H_ ≥ 40, P_GeH_ increases to its maximum value P_GeH_ = 4.3 at R_H_ = 75 while P_SiH_ reduces to P_SiH_ ≈ 0.22. It means that all Ge atoms have been mostly terminated by hydrogen in contrast to Si atoms. Thus we can clearly distinguish three regions: low R_H_ ≤ 20, where Si atoms are preferentially terminated with hydrogen while Ge atoms have shortage of hydrogen, medium region 20 ≤ R_H_ ≤ 40 where H-termination starts to change (P_SiH_ reduces and P_GeH_ slightly increases), and finally the region of high dilution R_H_ ≥ 40, where Ge atoms are mostly terminated by hydrogen but Si atoms do not have sufficient hydrogen. It would be reasonable to expect that for such “anti-correlation” between hydrogen terminations of Si and Ge atoms, for the low R_H_ region the defects expected would be due to Ge atoms not passivated by hydrogen. For a high level of H-dilution R_H_ ≥ 40, Ge atoms are completely terminated with hydrogen, while Si atoms have not enough hydrogen passivation and therefore would reveal themselves as defects (e.g., dangling bonds).

This next section is divided by subheadings. It provides a concise and precise description of the experimental results, their interpretation, as well as the experimental conclusions that can be drawn.

## 4. Discussion

### 4.1. Incorporation of Si, Ge in RF Versus LF PECVD

It is interesting to compare the growth rate of the films deposited with H-dilution. In reference [9] an increase in the growth rate is correlated with Ge content in both the gas phase and solid phase for Si-Ge films deposited with fixed H-dilution (R_H_ = 20). The films studied in [9] were deposited by low-frequency PECVD at fixed R_H_ = 10 with different Ge content in the gas. In this work, the films were grown by RF PECVD at low deposition temperature. The Ge relative content was equal to that of Si in the gas phase and both were fixed at the value 0.5 while R_H_ was varied. Thus, the conditions were different in this work and the reference [9]. If we compare the deposition rate observed in this work and that reported in [9] at “similar” R_H_ = 20, and relative Ge, Si content in gas phase 0.5, one can see that V_d_ = 0.9 A/s in this work (RF discharge) is less than V_d_ = 2.3 A/s (LF discharge) reported in [9]. However, this comparison does not allow the conclusion that the deposition rate in RF discharge is less than that in LF discharge due to the discharge power density in [9] where the LF discharge was remarkably higher (W = 120 mW/cm^2^) than that in RF discharge (W = 22 mW/cm^2^ in RF discharge). Additionally, the deposition temperature in [9] was higher (T_d_ = 300 °C) than the temperature used in this work (T_d_ = 160 °C). The deposition pressure is similar in both LF (P = 0.60 Torr) and RF (P = 0.55 Torr) experiments.

Preferential incorporation of Ge atoms was observed for film growth at R_H_ = 20 where the preferential factor for Ge was P_Ge_ = 6.44 even though the concentration of Ge and Si atoms was kept equal in the gas mixture. In this work, we observed also Ge preferential incorporation for R_H_ > 15 but the preferential factor changed with R_H_ from P_Ge_ = 1.2 at R_H_ = 15 to maximum P_Ge_ = 4.2 at R_H_ = 75. It is worth noting that despite the Ge preferential incorporation in the range of 15 < R_H_ < 80, the growth rate (following the trend of the Si incorporation) and hydrogen content (determined by SIMS) suggest that the growth rate is determined by Si atoms incorporation.

**Hydrogen content vs H dilution:** If we compare hydrogen content versus H-dilution determined by SIMS presented in Figure 3, it shows a decrease from [H]_sims_ = 0.25 at R_H_ = 10 to [H]_sims_ ≈ 0.15 at R_H_ = 30 and further, it stays constant with Si–H and Ge–H concentrations as a function of R_H_. We can also see another trend: increasing of H bonded content with R_H_ with a maximum at R_H_ = 70. Taking into account that the SIMS signal is determined by both chemically bonded hydrogen and hydrogen absorbed (in pores and on surface) rather than only for chemically bonded hydrogen as is the case of FTIR measurements it is reasonable to suggest that with an increase of R_H_, the total hydrogen content is reduced and the relative part of hydrogen-related to chemically bonded hydrogen is increased.

Let us compare our results on chemically bonded hydrogen distribution between Ge and Si atoms with those reported in the literature. In reference [9] the authors observed P_GeH_ = 0.45, 0.61, and 0.29 in the films deposited with hydrogen (R_H_ = 20), argon dilution and without dilution in LF PECVD, respectively. Data obtained in this work in the range of 10 ≤ R_H_ ≤ 40 in RF discharge P_GeH_ ≈ 0.5 are in good agreement with those reported in [9]. However, we studied also the films deposited with higher values of R_H_ from 40 to 80 and we observed an increase of P_GeH_ to 3.2 at R_H_ = 75–80. Thus we demonstrated that it is possible to control both Ge content in the solid film (from [Ge]_sol_ = 0.5 to 0.7) and hydrogen termination of Si and Ge atoms (from P_GeH_ = 0.5 to P_GeH_ = 3.2) by varying hydrogen dilution R_H_ from 10 to 80.

### 4.2. Reduction of Vd and Preferential Solid Incorporation of Ge Atoms

Because the PECVD deposition of thin films is a complex process, the final characteristics of the films vary from system to system and growth conditions have interdependence from parameters such as power density, substrate temperature, chamber pressure, etc. In this section, we discuss only the results based on three general processes: (i) Gas/plasma phase process, (ii) species transport process to the surface and (iii) solid growth process on the surface. In (i), increasing R_H_, while pressure and RF power are kept constant, means dilution of the silicon and germanium precursors decreasing the number of radicals available to be transported to the surface. Thus, V_d_ decreases with increase of R_H_ (Figure 4). On the other hand, the amount of hydrogen radicals is increased altering the reaction in the plasma. It is important to note that the binding energy of Ge–H (2.97 eV/bond) is lower than that of Si–H (3.2 eV/bond) [20], then, it is expected that decomposition of GeH_4_ will be faster than SiH_4_ and the Ge radicals will become an important factor for growth when both, silicon and germanium radicals are scarce. This explains the preferential solid incorporation of Ge atoms to the film when R_H_ is increased. In (ii), the sheath region of the glow discharge is responsible for transporting the species from plasma bulk to the film surface and this also results in ion bombardment. In consequence, the increase of R_H_ is expected to increase the number of H radicals for diffusion, sticking, and etching in (iii) [21], then the possible effects of hydrogen dilution include: (a) Etching of weak bonds with preferential etching of silicon over germanium atoms [22], (b) more hydrogen atoms sticking to superficial bonds decreasing the reactivity of the surface and (c) hydrogen atom diffusion into the bulk causing restructuration of the alloy network (for example, increasing the H termination). In this case, etching and sticking will contribute to the reduction of V_d_ and explain the preferential solid incorporation of Ge atoms in the solid phase (Figure 4 and Figure 5).

### 4.3. Preferential Ge–H Termination

According to Section 4.1, the increase of hydrogen dilution enhances hydrogen termination on the surface but also increases the implantation of hydrogen atoms due to ion bombardment [21]. In this case, hydrogen radicals provide additional energy to activate the reaction of hydrogen with silicon and germanium atoms on the surface. This factor is mainly important if the deposition process is at low deposition temperatures (T_d_ < 200 °C). On the other hand, the increase of preferential termination of Ge atoms with the increase of hydrogen dilution is explained by a Ge-dominated surface where the reactions of hydrogen activation are concentrated. This Ge-dominated surface is the result of an increase of germanium in the solid/gas phase and preferential etching of silicon atoms [22]. The high rate of Ge–H termination in silicon-germanium thin films has a strong correlation to stability against light exposure. It is important to note, then, that an increase of H dilution increases the amount of Ge atoms terminated by hydrogen (Figure 8), but reduces the amount of hydrogen in the solid phases (Figure 4). This may due to the extra energy provided by ion bombardment of H radicals on the surface that also stimulates the local desorption of hydrogen.

## 5. Conclusions

The effect of H-dilution in the range of R_H_ = 10 to 80 on the composition and hydrogen termination of Si and Ge atoms in Si_X_Ge_Y_:H_Z_ films deposited by RF PECVD at low deposition temperature (T_d_ = 160 °C) was investigated. Incorporation of Si and Ge atoms from the gas phase into the solid films depended strongly on H-dilution: reducing for Si and increasing for Ge atoms with an increase of R_H_ from 10 to 30. Preferential incorporation increased for Ge atoms (P_Ge_) and reduced for Si atoms (P_Si_) with an increase of R_H_. Maximum P_Ge_ = 4.2 is obtained in the films deposited at R_H_ = 70 while the maximum P_Si_ was P_Si_ = 1.3 at R_H_ = 10. This means that hydrogen dilution results in a significant increase of Ge atom incorporation.

H-termination of Si and Ge atoms studied by FTIR spectroscopy revealed that at low H-dilution (R_H_ = 10), hydrogen preferentially terminated Si atoms with a P_SiH_ = 3.2. Then in the range of 20 < R_H_ < 40, P_SiH_ decreases to the value P_SiH_ = 1.7. Further increase of H-dilution from R_H_ = 40 to 50 P_SiH_ results in a P_SiH_ = 0.5, staying without change up to R_H_ = 80. The H-termination of Ge atoms was practically not changed from the value R_GeH_ = 0.5 in the range of 10 < R_H_ < 40. Further increase of R_H_ from R_H_ = 40 to 80 resulted in an increase of P_GeH_ from 0.5 to 4.3 providing substantial preferential incorporation for Ge atoms (P_GeH_ = 4.3) in the films deposited at high hydrogen dilution (R_H_ = 80). From these observations and data analysis under the studied conditions we can draw the following conclusions:

(1) Dilution by hydrogen decreases the deposition rate due to factors such as the dilution of silicon and germanium precursors, etching of the silicon atoms, and reduction of the reactivity of the surface.
(2) For all films, preferential incorporation of Ge atoms (P_Ge_) is observed. This may be associated with the lower binding energy of Ge–H (2.97 eV/bond) than that of Si–H (3.2 eV/bond) which results in more germanium precursors in the plasma. The lower preferential incorporation coefficient of silicon atoms can be explained by the preferential etching of silicon atoms due to an increase of hydrogen radicals.
(3) Hydrogen preferentially terminates Ge atoms with an increase of the hydrogen dilution as a consequence of an increase of Ge atoms in the solid phase on the surface and an increase of surface energy provided by the hydrogen radicals.

## Appendix B

**Table A1:** Peak assignments in FTIR spectra of the films studied.

Sample	K [cm^−1^]	W [cm^−1^]	S [cm^−2^]	Bonding (cm^−1^) Ref. [23]
**R_H_ = 09**	608.2 ± 0.5	111 ± 1	(1.50 ± 0.03) × 10^5^	Ge–H (570) and Si–H (630) bending
	1883.2 ± 0.9	81 ± 3	(1.9 ± 0.1) × 10^4^	Ge–H (1880) stretching
	2015 ± 1	101 ± 3	(4.6 ± 0.2) × 10^4^	Si–H (2000) stretching
	2098 ± 2	69 ± 4	(1.5 ± 0.2) × 10^4^	SiH_2_ (2090–2140) stretching
**R_H_ = 20**	570 ± 5	73 ± 6	(4.9 ± 0.8) × 10^4^	Ge–H (570) bending
	616 ± 1	47 ± 5	(3.5 ± 0.9) × 10^4^	Si–H (630) bending
	664 ± 1	41 ± 1	(3.1 ± 0.2) × 10^4^	Si–H_2_ (670) wagging or Si–C stretching
	1883.2 ± 0.8	86 ± 4	(2.5 ± 0.2) × 10^4^	Ge–H (1880) stretching
	2013 ± 3	77 ± 4	(2.9 ± 0.2) × 10^4^	Si–H (2000) stretching
	2089 ± 6	85 ± 13	(1.5 ± 0.4) × 10^4^	SiH_2_ (2090–2140) stretching
**R_H_ = 30**	559 ± 1	40 ± 2	(1.9 ± 0.3) × 10^4^	Ge–H (570) bending
	608.2 ± 0.9	61 ± 5	(5.3 ± 0.4) × 10^4^	Ge–H (570) and Si–H (630) bending
	666.9 ± 0.8	45 ± 1	(3.8 ± 0.2) × 10^4^	Si–H_2_ (670) wagging or Si–C stretching
	1878 ± 2	65 ± 3	(3.9 ± 0.3) × 10^4^	Ge–H (1880) stretching
	2015 ± 1	67 ± 5	(3.9 ± 0.5) × 10^4^	Si–H (2000) stretching
	2093 ± 5	93 ± 8	(2.8 ± 0.3) × 10^4^	SiH_2_ (2090–2140) stretching or SiH–O
**R_H_ = 40**	571 ± 5	68 ± 5	(4.7 ± 0.9) × 10^4^	Ge–H (570) bending
	618 ± 2	54 ± 6	(4 ± 1) × 10^4^	Ge–H (570) and Si–H (630) bending
	1873 ± 2	69 ± 7	(4.5 ± 0.8) × 10^4^	Ge–H (1880) stretching
	2008 ± 1	64 ± 3	(4.4 ± 0.4) × 10^4^	Si–H (2000) stretching
	2096 ± 2	91 ± 8	(3.8 ± 0.6) × 10^4^	SiH_2_ (2090–2140) stretching or SiH–O
**R_H_ = 50**	579.2 ± 0.5	71 ± 1	(4.5 ± 0.1973) × 10^4^	Ge–H (570) bending
	667.3 ± 0.3	53.2 ± 0.9	(5.6 ± 0.1) × 10^4^	Si–H_2_ (670) wagging or Si–C
	1876.0 ± 0.2	18.2 ± 0.5	(5.0 ± 0.2) × 10^3^	Ge–H (1880) stretching
	2002.1 ± 0.3	22.0 ± 0.8	(3.5 ± 0.1) × 10^3^	Si–H (2000) stretching
	2030.2 ± 0.7	23 ± 1	(1.8 ± 0.1) × 10^3^	GeH_3_ (2050–2060) stretching
**R_H_ = 60**	578.1 ± 0.9	92 ± 3	(3.0 ± 0.2) × 10^4^	Ge–H (570) bending
	1875.2 ± 0.5	38 ± 1	(1.45 ± 0.05) × 10^4^	Ge–H (1880) stretching
	2009 ± 1	33 ± 2	(5.7 ± 0.6) × 10^3^	Si–H (2000) stretching
	2031.9 ± 0.7	17 ± 1	(1.7 ± 0.3) × 10^3^	GeH_3_ (2050–2060) stretching
	2066.1 ± 0.6	23 ± 2	(1.5 ± 0.2) × 10^3^	GeH_3_ (2050–2060) stretching
**SiGe:H** **R_H_ = 70**	565.5 ± 0.9	43 ± 1	(2.8 ± 0.1) × 10^4^	Ge–H (570) bending
	611.8 ± 0.5	40 ± 1	(3.4 ± 0.1) × 10^4^	Si–H (630) bending
	667.6 ± 0.2	43.6 ± 0.6	(6.3 ± 0.1) × 10^4^	Si–H_2_ (670) wagging or Si–C stretching
	1877.6 ± 0.4	34 ± 2	(1.9 ± 0.1) × 10^4^	Ge–H (1880) stretching
	2032.6 ± 0.5	55 ± 1	(1.78 ± 0.09) × 10^4^	Si–H y GeH_3_Stretching
	2130.3 ± 0.7	72 ± 2	(2.2 ± 0.1) × 10^4^	SiH_3_ (2120–2140)Bending
	2178 ± 1	15 ± 3	(1.0 ± 0.2) × 10^3^	SiH-O_2_ (2160) cluster
**SiGe:H** **R_H_ = 75**	559 ± 1	46 ± 3	(3.1 ± 0.2) × 10^4^	Ge–H (570) bending
	610 ± 0.8	44 ± 2	(4.0 ± 0.2) × 10^4^	Si–H (630) bending
	666 ± 0.4	41 ± 1	(4.9 ± 0.1) × 10^4^	Si–H_2_ (670) wagging or Si–C stretching
	1879 ± 0.3	41.1 ± 0.9	(2.17 ± 0.05) × 10^4^	Ge–H (1880) stretching
	2002.1 ± 0.6	23 ± 1	(4.8 ± 0.4) × 10^3^	Si–H (2000) stretching
	2036.9 ± 0.5	33 ± 1	(9.2 ± 0.5) × 10^3^	GeH_3_ (2050–2060) stretching
	2089 ± 1	39 ± 5	(4.0 ± 0.7) × 10^3^	SiH_2_ (2090–2140) stretching
	2143 ± 1	42 ± 6	(5.7 ± 0.8) × 10^3^	SiH_3_ (2120−2140)Bending
	2179 ± 1	20 ± 3	(2.0 ± 0.5) × 10^3^	SiH–O_2_ (2160) cluster
**SiGe:H** **R_H_ = 80**	560.9 ± 0.4	42 ± 1	(2.9 ± 0.1) × 10^4^	Ge–H (570) bending
	611.4 ± 0.3	40.3 ± 0.9	(3.8 ± 0.1) × 10^4^	Si–H (630) bending
	667.4 ± 0.3	36 ± 1	(1.8 ± 1.2) × 10^4^	Si–H_2_ (670) wagging or Si–C stretching
	1880.4 ± 0.4	41 ± 1	(1.61 ± 0.04) × 10^4^	Ge–H (1880) stretching
	2000.2 ± 0.9	25 ± 2	(4.1 ± 0.8) × 10^3^	Si–H (2000) stretching
	2037.7 ± 0.7	36 ± 3	(8.7 ± 0.7) × 10^3^	GeH_3_ (2050–2060) stretching
	2079 ± 1	26 ± 3	(3.1 ± 0.7) × 10^3^	SiH_2_ (2090–2140) stretching
